# Race-ethnic differences in the association of genetic loci with HbA_1c_ levels and mortality in U.S. adults: the third National Health and Nutrition Examination Survey (NHANES III)

**DOI:** 10.1186/1471-2350-13-30

**Published:** 2012-04-27

**Authors:** Jonna L Grimsby, Bianca C Porneala, Jason L Vassy, Quanhe Yang, José C Florez, Josée Dupuis, Tiebin Liu, Ajay Yesupriya, Man-Huei Chang, Renee M Ned, Nicole F Dowling, Muin J Khoury, James B Meigs

**Affiliations:** 1 General Medicine Division, Massachusetts General Hospital, Boston, MA, USA; 2 Harvard Medical School, Boston, MA, USA; 3 Office of Public Health Genomics, Centers for Disease Control and Prevention, Atlanta, GA, USA; 4 Diabetes Unit and Center for Human Genetic Research, Massachusetts General Hospital, Boston, MA, USA; 5 Program in Medical and Population Genetics, Broad Institute, Cambridge, MA, USA; 6 Boston University School of Public Health, Boston, MA, USA; 7 National Heart, Lung, and Blood Institute's Framingham Heart Study, Framingham, MA, USA; 8 Meta-analysis of Glucose and Insulin related traits Consortium, a, a

## Abstract

**Background:**

Hemoglobin A_1c_ (HbA_1c_) levels diagnose diabetes, predict mortality and are associated with ten single nucleotide polymorphisms (SNPs) in white individuals. Genetic associations in other race groups are not known. We tested the hypotheses that there is race-ethnic variation in 1) HbA_1c_-associated risk allele frequencies (RAFs) for SNPs near *SPTA1, HFE, ANK1, HK1, ATP11A, FN3K, TMPRSS6*, G*6PC2, GCK, MTNR1B*; 2) association of SNPs with HbA_1c_ and 3) association of SNPs with mortality.

**Methods:**

We studied 3,041 non-diabetic individuals in the NHANES (National Health and Nutrition Examination Survey) III. We stratified the analysis by race/ethnicity (NHW: non-Hispanic white; NHB: non-Hispanic black; MA: Mexican American) to calculate RAF, calculated a genotype score by adding risk SNPs, and tested associations with SNPs and the genotype score using an additive genetic model, with type 1 error = 0.05.

**Results:**

RAFs varied widely and at six loci race-ethnic differences in RAF were significant (p < 0.0002), with NHB usually the most divergent. For instance, at *ATP11A*, the SNP RAF was 54% in NHB, 18% in MA and 14% in NHW (p < .0001). The mean genotype score differed by race-ethnicity (NHW: 10.4, NHB: 11.0, MA: 10.7, p < .0001), and was associated with increase in HbA_1c_ in NHW (β = 0.012 HbA_1c_ increase per risk allele, p = 0.04) and MA (β = 0.021, p = 0.005) but not NHB (β = 0.007, p = 0.39). The genotype score was not associated with mortality in any group (NHW: OR (per risk allele increase in mortality) = 1.07, p = 0.09; NHB: OR = 1.04, p = 0.39; MA: OR = 1.03, p = 0.71).

**Conclusion:**

At many HbA_1c_ loci in NHANES III there is substantial RAF race-ethnic heterogeneity. The combined impact of common HbA_1c_-associated variants on HbA_1c_ levels varied by race-ethnicity, but did not influence mortality.

## Background

The prevalence of type 2 diabetes (T2D) is not equal among race-ethnic groups in the United States, with a prevalence of 12.8% in non-Hispanic blacks (NHB), 8.4% in Mexican Americans (MA), and 6.6% in non-Hispanic whites (NHW) aged 20 yrs or older [1]. Diabetes-related complications also differ between race-ethnicities [2] and there is greater impact of diabetes on life-years in minority groups [3]. Race-ethnic differences in environmental exposures and health care experiences [4] likely influence different outcomes for people with diabetes, but genetic differences may also play an important role. Despite recent advances in the study of T2D genetics, relatively little is known about how race-ethnic genetic differences contribute to inter-race variability in diabetes risk or diabetes-related traits.

Percent HbA_1c_ (glycated hemoglobin) is an informative trait for diabetes diagnosis and management. It is accurate in quantifying chronic glycemic exposure of erythrocytes for the preceding 2–3 months, and there is a robust correlation between HbA_1c_ levels and occurrence of diabetes complications [5,6]. Recently, MAGIC (Meta-analyses of Glucose and Insulin related traits Consortium) identified ten genetic loci associated with HbA_1c_[7]. The ten loci associated included three loci in or near genes likely involved in glycemic control pathways: *G6PC2* (glucose-6-phosphatase catalytic subunit 2, MIM 608058), *GCK* (glucokinase, maturity onset diabetes of the young (MODY) 2, MIM 138079), and *MTNR1B* (melatonin receptor 1B, MIM 600804) and seven loci in or near genes likely to be involved in erythrocyte biology, including *SPTA1* (spectrin alpha erythrocytic 1, MIM 182860), *HFE* (hemochromatosis, MIM 235200), *ANK1* (ankyrin 1, MIM 612641), *HK1* (hexokinase 1, MIM 142600), *APT11A* (ATPase Class VI, type 11A, MIM 605868), *FN3K* (fructosamine 3 kinase, MIM 608425), and *TMPRSS6* (transmembrane protease serine 6, MIM 609862). Since MAGIC only included individuals of European ancestry, nothing is known about the impact of these risk alleles on HbA_1c_ levels in non-European-ancestry populations.

Given the selection pressure by infectious diseases such as malaria on some erythrocyte-related genes in African populations [8-10] and the influence of erythrocyte genes on HbA_1c_[7,11], we hypothesized that risk alleles at HbA_1c_-associated loci may have substantial race-ethnic frequency variation and that associations with HbA_1c_ levels may also differ by race. Furthermore, since elevated HbA_1c_ is associated with risk of cardiovascular disease or mortality [12-19], we hypothesized that an association between HbA_1c_-associated SNPs and mortality may exist and there may be race-ethnic differences in this association. Using 11 confirmed HbA_1c_-associated SNPs at ten loci [7], we compared NHB, MA, and NHW individuals from NHANES (National Health and Nutrition Examination Survey) III to test the hypotheses that there is significant race-ethnic variation in HbA_1c_ risk (HbA_1c_-raising) allele frequency, risk-allele association with HbA_1c_ levels and risk-allele association with mortality.

## Methods

### Study subjects from the third national health and nutrition examination survey

NHANES III was a nationally representative sample of the non-institutionalized civilian U.S. population collected using stratified multistage probability sampling. NHANES participants underwent a physical examination, phlebotomy, and a household interview [20]. This study was limited to non-diabetic patients (aged 20 or older) with 8–23 hours of fasting prior to blood sampling. Blood from NHANES III Phase II (1991–1994) participants aged 12 or older were used to generate Epstein-Barr transformed lymphocyte cell lines for DNA extraction. Mortality data (death within a mean of 13.5 years of follow-up) were merged from the NHANES III mortality-linked data file. Race-ethnic group was assigned based on self-report. The survey asked each subject to categorize his/her race as “white,” “black,” or “other” and his/her ethnicity as “Mexican-American,” “other Hispanic,” or “not Hispanic.” Of 3,894 individuals with complete data for analysis, we excluded 149 who were not of NHB, MA or NHW race-ethnicity and 704 with diabetes (293 NHW, 167 NHB and 244 MA), leaving 901 NHB, 909 MA, and 1,231 NHW individuals in the analysis. Written informed consent was obtained from all subjects and this study was approved by the National Center for Health Statistics (NCHS) Ethics Review Board.

### Diabetes definition and HbA_1c_ measures

Individuals with diabetes were excluded to avoid the confounding effects of treatment on HbA_1c_. We defined diabetes as a fasting plasma glucose ≥ 7.0 mmol/L, report of a diagnosis of diabetes or use of hypoglycemic medications. HbA_1c_ levels were measured using HPLC (Bio-Rad DIAMAT glycosylated hemoglobin analyzer system) [21].

### SNP genotyping and allele frequencies

Genotyping was performed using Sequenom iPLEX. We genotyped 11 SNPs at ten loci shown among white non-diabetic individuals in MAGIC to have genome-wide significant association with HbA_1c._[7] We used SNP rs282606 as a proxy for *ATP11A* rs7998202 (CEU r^2^ = 1.0), SNP rs10830956 as a proxy for *MTNR1B* rs1387153 (CEU r^2^ = 1.0), and rs2022003 as a proxy for *SPTA1* rs2779116 (CEU r^2^ = 0.927) [r^2^ for ASW and MEX populations not available]. The minimum call rate for genotyping was 95%. Allele frequencies of all SNPs were in Hardy Weinberg Equilibrium (HWE) based on National Center for Health Statistics standards (HWE rejected if *p* < 0.01 in ≥ 2 or more race-ethnic groups). We compared NHANES observed allele frequencies with those available from HapMap (http://hapmap.ncbi.nlm.nih.gov/, Release 27, Phases II and III, NCBI build 36), comparing NHW with CEU (Utah residents with Northern and Western European ancestry from the CEPH collection), NHB with ASW (African ancestry in Southwest USA), and MA with MEX (Mexican ancestry in Los Angeles, California).

### Genotype risk score

We calculated a genotype risk score to test the collective association with HbA_1c_ of 11 SNPs at 10 loci (2 uncorrelated SNPs at *ANK1*). We assumed that each SNP was associated with HbA_1c_ based on previous association results in whites, despite potential ancestral differences in NHB or MA in linkage disequilibrium (LD) patterns [22]. Since we did not know the effect size of the MAGIC SNPs in non-white populations, we did not apply SNP-specific weights to account for SNP-specific differences in effect on HbA_1c_, but simply summed the presence of 0, 1, or 2 risk alleles carried by individuals at each SNP. In addition to the 11-SNP GRS, we also performed a secondary analysis using an eight SNP “non-glycemic” risk score by excluding the three glycemic loci (G*6PC2, GCK, MTNR1B*) for score calculation.

### Statistical analyses of association

We stratified the analysis by race-ethnicity (NHB, MA, and NHW) and to estimate rates and proportions within groups used weights to account for sampling probabilities using methods previously described [23]. *P*-values for differences across race-ethnic groups were calculated using Satterthwaite adjusted- F statistics for continuous variables and chi-square tests for categorical variables. To estimate the significance of differences in allele frequencies across groups we used Fisher’s Exact tests.

To investigate the relationship between SNPs and HbA_1c_ level we used linear regression and an additive genetic model adjusted for age and sex. We included one SNP at a time in the models for individual SNP associations with HbA_1c_, with genotypes coded as 0, 1 or 2 depending on the number of HbA_1c_-raising alleles present. To study the collective effect of the 11 SNPs on HbA_1c_ we used linear regression adjusted for age and sex, totaled the number of risk alleles at all 11 SNPs to calculate a risk score, and tested associations of a per-risk-allele increase in genotype risk score with HbA_1c_. We calculated the adjusted model R^*2*^ with and without the genotype risk score for each group to determine the percent variance in HbA_1c_ explained by genetic effects. The same procedure was carried out for the 8 SNP “non-glycemic” risk score, as well as for genetic associations with mortality (percent dead as of 13.5 years post-baseline exam). To determine if a significant genetic risk score x ethnicity interaction effect on HbA_1c_ exists, we also applied the following linear regression model on the whole sample: Hba_1c_ level (outcome) = sex, age, genetic risk score, ethnicity, genetic risk score x ethnicity interaction. For tests of association with mortality we used logistic regression to estimate the odds of mortality with per-risk-allele increase in HbA_1c_. For analysis of mortality, Cox models yielded similar results to logistic regression, so Cox model results are not shown. We also applied the following logistic regression model on the whole sample: mortality (outcome) = sex, age, GRS, ethnicity, GRS x ethnicity interaction. For the analyses we used SUDAAN (version 10.0) [24] and SAS (version 9.2, SAS Institute Inc, Cary, NC). We considered p values less than 0.05 to indicate statistical significance, based on one test per previously established SNP at each locus for each hypothesis (SNP is associated with HbA1c; SNP is associated with mortality).

### Linkage disequilibrium, signatures of population differentiation and natural selection at HbA_1c_-associated loci

To evaluate inter-ethnic differences in LD near the SNPs, we examined 500 kb around each SNP (HapMap Release 27, Build 36, phases II and III) for four populations (CEU, YRI, ASW, and MEX). Using Haploview version 4.2, [25] we counted the number of “Gabriel” LD regions (based on confidence intervals) [26] in that region for each population. We investigated natural selection around the ten loci using Haplotter [27] and HapMap Phase II data. Standardized Integrated Haplotype Score (iHS) (a statistic based on differential LD around positively selected alleles that compares haplotype length with ancestral allele versus derived allele to detect positive selection) [27], Fay and Wu’s H + statistic (a measure used to scan a region for allele frequencies that are skewed from the neutral model) [28] and the Fixation Index (F_ST_) (a statistic using allele frequencies to measure genetic divergence between subpopulations) [29] were obtained through Haplotter SNP queries spanning 2 Mb regions at each locus.

## Results

### Characteristics of participants

NHW individuals were older, had lower BMI and lower mean HbA_1c_ than did NHB and MA individuals (global *p* values all <0.0001, Table 1).

**Table 1:** Characteristics of participants by race-ethnicity, Third National Health and Nutrition Examination Survey (NHANES III)

	**Sample weighted distribution by race-ethnicity**^**1**^			
	**Non-Hispanic White**	**Non-Hispanic Black**	**Mexican American**	
**Characteristics**	**(n = 1231)**	**(n = 901)**	**(n = 909)**	**p-value**^**2**^
**HbA**_**1c**_**(%)** (SE)	5.22 (0.015)	5.36 (0.022)	5.30 (0.016)	<.0001
**Age (years)**				
Mean (SE)	43.7 (0.52)	39.6 (0.52)	35.6 (0.45)	<.0001
**Sex**				
Male, % (95% CI)	48.6 (45.1–52.2)	44.1 (40.6–47.7)	53.3 (49.6–57.0)	
Female, % (95% CI)	51.4 (47.9–54.9)	55.9 (52.3–59.4)	46.7 (43.0–50.4)	0.2713
**BMI (kg/m**^**2**^**)**				
>25 (95% CI)	45.1 (41.6–48.6)	33.4 (30.0–36.7)	32.4 (28.9–35.9)	
25 to >30 (95% CI)	34.3 (30.9–37.8)	36.3 (32.8–39.7)	41.6 (37.9–45.3)	
> 30 (95% CI)	20.6 (17.9–23.4)	30.4 (27.2–33.6)	26.1 (22.7–29.4)	<.0001

*CI* = confidence interval; *Std Dev*: standard deviation; *SE*: standard error; *BMI*: Body Mass Index; *NHW*: non-Hispanic white; *NHB*: Non-Hispanic black; *MA*: Mexican American.

1. For continuous variables, means were adjusted for age and sex.

2. p-values were tests for the difference across race-ethnicity based on Satterthwaite adjusted-F statistics for continuous variables and X^2^ for categorical variables.

### Risk allele frequencies of HbA_1c_-associated variants

**Figure 1:**
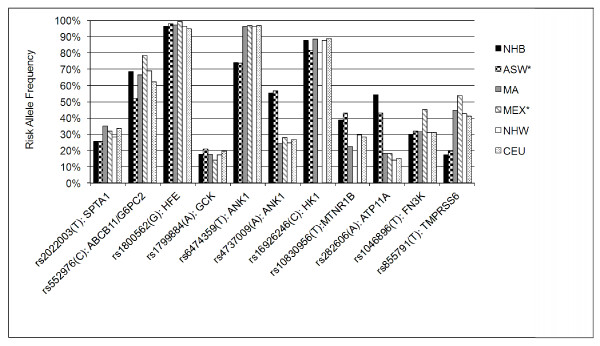
**Risk allele frequencies of 11 HbA**_**1c**_**-associated SNPs.***NHB*: Non-Hispanic black NHANES III; *MA*: Mexican American NHANES III; *NHW*: Non-Hispanic white NHANES III; *ASW*: African ancestry in Southwest USA HapMap; *MEX*: Mexican ancestry in Los Angeles, California HapMap; *CEU*: Utah residents with Northern and Western European ancestry from the CEPH collection HapMap. *ASW frequencies were missing at *ANK1* (rs6474359), *HK1* and *MTNR1B* loci and YRI frequencies were substituted (YRI: Yoruba in Ibadan, Nigeria). MEX frequencies were missing at *HK1* and *MTNR1B* loci.

Risk allele frequencies across the 11 loci varied widely within the three race-ethnic groups ( Additional file 1 Table S1). Six out of 11 HbA_1c_–associated SNPs had risk allele frequencies that differed significantly across race-ethnic groups (Fisher’s *p* <0.0002). At five of these six loci, risk allele frequency of NHB was most divergent, including SNPs near *ANK1* (two uncorrelated SNPs), *MTNR1B*, *ATP11A/TUBGCP3* and *TMPRSS6*. At the SNP near *SPTA1*, risk allele frequency differed most in MA. The HbA_1c_ -raising allele was the minor (less frequent) allele in all three ethnic groups for SNPs near *SPTA1*, *GCK*, *MTNR1B*, *FN3K*, and *TMPRSS6*. The HbA_1c_ –raising allele was the major (more frequent) allele at SNPs near *ABCB11*, *HFE*, *ANKI* (rs6474359) and *HK1*. At two loci, *ATP11A*, and *ANK1* (rs4737009), the HbA_1c_ –raising allele was the minor allele in NHW and MA, but the major allele in NHB ( Additional file 1: Table S1). Risk allele frequencies observed in this study and those available from HapMap were generally similar, although at some loci minor dissimilarity with HapMap was observed in NHB and MA cohorts (Figure 1; Additional file 1: Table S2).

### SNP associations with HbA_1c_

Though single-SNP associations are underpowered ( Additional file 1: Table S3), we did observe that in NHW, eight of the 11 SNPs in NHW were consistent with Soranzo et al. (2010) in having a positive risk effect on HbA_1c_ levels, with three of the SNPs used in our analysis (rs282606 [*ATP11A*], rs10830956 [*MTNR1B*], and rs2022003 [*SPTA1*]) serving as proxies for those in the MAGIC study. Beta coefficients were negative for three, three and four of the 11 SNPs in NHW, NHB and MA groups, respectively, but corresponding SNPs did not generate significant associations (Table 2). Three out of 11 HbA_1c_-associated SNPs had nominally significant (*p* < 0.05) associations with HbA_1c_ levels in at least one of the three race-ethnic groups, but altogether only four of the 33 possible associations (11 SNPs x three race-ethnic groups) were significant (*p* < 0.05). No significant associations were observed in NHB. Two HbA_1c_-SNPs produced a significant association only in NHW (both SNPs at *ANK1*), and one produced a significant association in both NHW and MA (rs855791 near *TMPRSS6*).

**Table 2:** **Regression coefficients of 11 HbA**_**1c**_**-associated SNPs on HbA**_**1c**_**levels by race-ethnicity, Third National Health and Nutrition Examination Survey (NHANES III)**

			**Non-Hispanic White**		**Non-Hispanic Black**		**Mexican American**		**p-value for heterogeneity**^**3**^
**SNPs**	**Chr.**	**Nearest Gene**	**β**^**1**^**(SE)**	**p-value**^**2**^	**β**^**1**^**(SE)**	**p-value**^**2**^	**β**^**1**^**(SE)**	**p-value**^**2**^	
rs2022003	1	*SPTA1*	0.021 (0.02)	0.34	0.024 (0.03)	0.48	0.038 (0.02)	0.09	0.75
rs552976	2	*ABCB11/G6PC2*	0.030 (0.02)	0.13	0.015 (0.03)	0.64	0.011 (0.02)	0.62	0.76
rs1800562	6	*HFE*	0.022 (0.06)	0.73	0.1041 (0.07)	0.14	0.005 (0.10)	0.96	0.27
rs1799884	7	*GCK*	-0.003 (0.03)	0.91	-0.031 (0.04)	0.40	0.041 (0.03)	0.13	0.78
rs4737009	8	*ANK1*	0.047 (0.02)	0.03	0.038 (0.03)	0.20	0.052 (0.03)	0.06	0.99
rs6474359	8	*ANK1*	0.1024 (0.05)	0.03	0.026 (0.04)	0.49	-0.002 (0.06)	0.97	0.41
rs16926246	10	*HK1*	-0.050 (0.03)	0.08	0.036 (0.04)	0.37	-0.005 (0.03)	0.87	0.71
rs10830956	11	*MTNR1B*	-0.010 (0.02)	0.62	-0.011 (0.03)	0.74	0.030 (0.03)	0.24	0.38
rs282606	13	*ATP11A /*	0.037 (0.03)	0.17	0.019 (0.03)	0.53	-0.015 (0.03)	0.60	0.90
rs1046896	17	*FN3K*	0.014 (0.02)	0.47	-0.034 (0.03)	0.24	-0.021 (0.02)	0.35	0.23
rs855791	22	*TMPRSS6*	0.048 (0.02)	0.01	0.025 (0.04)	0.49	0.050 (0.02)	0.02	0.52

*SE*: standard error; *Chr*.: chromosome.

1. β-coefficients (SE) (change in HbA_1c_ per SNP risk allele) of linear regression models adjusted for age and sex.

2. p-values for β-coefficients based on Satterthwaite adjusted-F test.

3. p-values for difference in β-coefficients acris race-ethnic groups.

### Combined associations of 11 HbA_1c_ SNPS with HbA_1c_

The mean 11-SNP genotype scores (actual scores ranged from 1–18) were 11.0 (± 0.09 [SE]) in NHB, 10.7 (± 0.08) in MA and 10.4 (± 0.07) in NHW, (*p* value for global difference across race-ethnicity < 0.0001, Table 3). Median genetic risk scores (unweighted) were 11.0 (SD =2.2), 11.0 (SD =2.3) and 11.0 (SD =2.0) in NHW, NHB and MA, respectively, with distributions of genetic risk scores negatively skewed toward a lower score in all three ethnic groups. The per-risk allele increase in the score was significantly associated with HbA_1c_ levels in NHW and MA, but not NHB. When comparing the top and bottom 10% of the genotype score distribution for each race-ethnic group, the smallest difference in HbA_1c_ was observed in NHW (NHW: 0.49%; NHB: 0.56%; MA: 0.54%). The genotype score explained very little of the variance in HbA_1c_ levels in NHB (0.0005%) compared with NHW (0.0016%) and MA (0.0121%). Variance explained in NHW is comparable to the previously published value [7]. We observed no significant genetic risk score x ethnicity interaction on HbA_1c_ level (p = 0.68).

**Table 3:** **Association of HbA**_**1c**_**with the 11 SNP genetic risk score by race-ethnicity, Third National Health and Nutrition Examination Survey (NHANES III)**

**Race/ethnicity**	**β (SE)**^**1**^	**p-value**^**2**^	**Sample N**	**Genetic Risk Score (SE)**^**4**^	****R**^**2 **^**Without Score**^**3**^**	**R**^**2**^**With Score**^**3**^	**R**^**2**^** Difference**	**HbA**_**1c**_**(%) difference top - bottom 10% of score distribution**
**Non-Hispanic White**								
**HbA**_**1c**_**(%)**	0.012 (0.006)	0.04	1231	10.42 (0.07)	0.214	0.218	0.004	0.49
**Non-Hispanic Black**								
**HbA**_**1c**_**(%)**	0.007 (0.008)	0.39	901	10.95 (0.09)	0.095	0.096	0.001	0.56
**Mexican Americans**								
**HbA**_**1c**_**(%)**	0.021 (0.008)	0.005	909	10.67 (0.08)	0.131	0.168	0.037	0.54

*HbA*_*1c*_: Hemoglobin A_1c_ (glycated hemoglobin); *SE*: standard error.

1. β-coefficients (SE) for the per-risk allele increase in HbA_1c_ (%) from linear regression models fro the risk score adjusted for age and sex.

2. p-values for β-coefficients based on Satterthwaite adjusted-F test.

3. Adjusted R^2^ for regression models with and without (age and sex only) weighted genetic risk score.

4. p = <.0001 for the global difference in weighted genetic risk score across race-ethnic groups.

### Combined associations of eight non-glycemic SNPs with HbA_1c_

The mean “non-glycemic” 8-SNP genotype scores (actual scores ranged from 4–15) were 8.80 (± 0.06[SE]) in NHB, 8.72 (± 0.06) in MA and 8.41(± 0.06) in NHW, (*p* value for global difference across race-ethnicity < 0.0001) ( Additional file 1: Table S4). The per-risk allele increase in the score was significantly associated with HbA_1c_ levels in NHW, but not in NHB and MA.

### Association of 11 HbA_1c_ SNPs with mortality

Mortality rates differed between race-ethnic groups (Table 4) with a higher mortality rate observed in NHB (19.4%) compared with NHW (12.8%) and MA (14.5%). The 11-SNP genotype score was not associated with mortality in any race-ethnic group. We observed no significant genetic risk score x ethnicity interaction on mortality (p=0.62). Power calculations for the mortality analysis are provided in Additional file 1: Table S5.

**Table 4:** Association of 11 SNPs and the Genotype Score with mortality by race-ethnicity, Third National Health and Nutrition Examination Survey (NHANES III)

			**Mortality at 13.5 years**								
			**with 0, 1, or 2 Risk Allele**								
**Race-ethnicity**	**SNPs**	**N**	**0**	**1**	**2**	**SNP per-risk alelle increase in mortality (OR) (95% CI)**	**p-value**^**1**^	**mortality events**	**Age and race standardized weighted mortality (%) per 1000 person-years (95% CI)**	**11 SNP Genotype Score per-risk alelle increase in mortality (OR) (95% CI)**	**p-value**^**3**^
								143	12.8 (11.0–14.6)	1.07 (0.99–1.16)	0.09
Non-Hispanic White	rs2022003	1,226	52.8	39.1	8.1	1.12 (0.81–1.55)	0.48				
	rs552976	1,192	9.9	41.5	48.6	0.93 (0.68–1.27)	0.68				
	rs1800562	1,204	0.1	8.0	91.9	1.07 (0.45–2.55)	0.88				
	rs1799884	1,207	69.2	27.6	3.3	0.98 (0.68–1.40)	0.90				
	rs4737009	1,183	57.7	35.1	7.2	0.97 (0.65–1.47)	0.91				
	rs6474359	1,174	0.1	6.8	93.1	0.91 (0.48–1.69)	0.76				
	rs16926246	1,206	2.5	20.7	76.8	1.34 (0.88–2.05)	0.17				
	rs10830956	1,173	49.3	42.9	7.9	1.14 (0.79–1.65)	0.48				
	rs282606	1,183	74.4	22.9	2.7	0.85 (0.58–1.22)	0.37				
	rs1046896	1,205	47.4	42.9	9.7	1.11 (0.80–1.54)	0.52				
	rs855791	1,183	33.2	48.3	18.5	1.14 (0.81–1.62)	0.45				
								100	19.4 (15.8–22.9)	1.04(0.95–1.14)	0.39
Non-Hispanic Black	rs2022003	869	55.2	39.2	5.6	1.40 (0.97–2.04)	0.07				
	rs552976	882	10.2	42.8	47.0	0.96 (0.67–1.39)	0.84				
	rs1800562	887	0.1	7.1	92.8	2.81 (1.11–7.13)	0.03				
	rs1799884	888	68.7	28.2	3.1	0.96 (0.65–1.43)	0.86				
	rs4737009	861	19.0	51.7	29.3	1.11 (0.77–1.59)	0.57				
	rs6474359	853	6.4	38.6	55.1	0.73 (0.51–1.03)	0.08				
	rs16926246	885	1.6	19.7	78.7	1.13 (0.66–1.92)	0.66				
	rs10830956	832	37.7	47.0	15.3	1.18 (0.84–1.67)	0.34				
	rs282606	859	20.2	50.8	29.1	0.93 (0.68–1.27)	0.63				
	rs1046896	888	50.2	40.9	8.9	1.17 (0.85–1.63)	0.33				
	rs855791	857	70.2	25.6	4.2	0.93 (0.63–1.38)	0.73				
								55	14.5 (12.0–16.9)	1.03 (0.90–1.18)	0.71
Mexican American	rs2022003	896	41.9	45.6	12.5	0.96 (0.64–1.44)	0.84				
	rs552976	890	13.6	41.5	44.8	0.99 (0.67–1.46)	0.97				
	rs1800562	904	0.1	4.8	95.1	1.30 (0.44–3.85)	0.63				
	rs1799884	904	68.7	27.3	4.0	0.85 (0.54–1.36)	0.50				
	rs4737009	884	57.6	37.1	5.2	1.33 (0.88–2.02)	0.18				
	rs6474359	883	0.2	6.8	93.0	0.85 (0.35–2.03)	0.71				
	rs16926246	904	1.3	19.4	79.3	2.04 (1.02–4.08)	0.04				
	rs10830956	874	59.7	33.8	6.5	0.90 (0.57–1.40)	0.64				
	rs282606	882	67.6	28.4	4.0	0.68 (0.41–1.12)	0.13				
	rs1046896	904	47.3	43.6	9.2	1.06 (0.70–1.60)	0.78				
	rs855791	878	30.2	50.7	19.2	0.95 (0.64–1.40)	0.80				

*SNP*: single nucleaotide polymorphism; *OR*: odds ratio; *CI*: confidence interval.

1. p-value associated with the OR for the per-risk allele increase in mortality for each SNP.

2. p=<.0001 for the global difference in mortality across race-ethnic groups.

3. p-value associated with the OR for the per-risk allele increase in mortality for the genotype risk score.

### Linkage disequilibrium at HbA_1c_-associated loci

There were consistently fewer LD regions in the CEU population compared to YRI at every locus (YRI:CEU): *SPTA1* 42:25; *ABCB11/G6PC2* 49:27; *HFE* 31:17; *GCK* 28:21; *ANK1* (rs41668351) 35:29; *ANK1* (rs41749562) 30:25; *HK1* 45:38; *MTNR1B* 39:22; *FN3K* 42:27; *TMPRSS6* 78:52; *ATP11A/TUBGCP3* 49:31 ( Additional file 1: Table 6). ASW, which represents a population with African ancestry in the southwestern United States, only had higher numbers of LD regions compared to CEU in two out of 11 regions, possibly due to lower coverage of ASW compared to CEU (and YRI) in HapMap Release 27.

### Evidence of population differentiation and natural selection at HbA_1c_-associated loci

Fay and Wu’s H + was highly skewed at two loci (*HK1* and *ATP11A)* in CEU ( Additional file 1: Table S7). Integrated haplotype scores (iHS) were not highly negative or positive at these SNPs, as would be characteristic for regions undergoing recent natural selection. F_ST_, a measure of the amount of allelic fixation due to drift, was greater than 15% at *ANK1* and *ATP11A* in both CEU and YRI, suggesting population differentiation at these loci [29]. Haplotter queries by gene did not reveal evidence of natural selection directly at the genes queried, but evidence of natural selection was observed within a 2 Mb region of *ABC11/G6PC2* and *TMPRSS6* for CEU and YRI, respectively.

## Discussion

Genome-wide association studies of HbA_1c_ levels in cohorts of white individuals of European ancestry revealed a combination of glycemic and non-glycemic biological influences on HbA_1c_, with three loci associated with HbA_1c_ in or near genes likely involved in glycemic control pathways and seven loci associated with HbA_1c_ in or near genes likely to be involved in erythrocyte biology [7]. In this study we found that in the nationally representative NHANES III sample of US adults, heterogeneity in risk allele frequencies exists across race-ethnic groups for six of these HbA_1c_-associated SNPs. Five SNP risk allele frequencies in NHB were significantly lower or higher than the other two groups. Risk allele frequencies observed in NHANES III were generally consistent with frequencies of comparable populations available in HapMap, suggesting that HapMap and NHANES III can be considered representative of each other at these SNPs at least with respect to white, African American and Mexican American race-ethnic populations. An 11-HbA_1c_- associated SNP genotype score was subtly different by race-ethnicity and was associated with increase in HbA_1c_ in NHW and MA but not NHB. The 11-SNP genotype score was not significantly associated with mortality in any group.

There are several potential sources for the inter-race-ethnic heterogeneity of SNP and genotype risk score associations with HbA_1c_ that we observed. One potential source of heterogeneity is race-specific selection acting on erythrocyte-related loci that influence HbA_1c_. Variants in the β- hemoglobin gene (*HBB*), for example, produce abnormal erythrocytes that can affect HbA_1c_ levels [30] but are protective against malaria and are thus maintained in populations and found at highest frequencies in regions historically exposed to this disease like Africa and India [31]. Rare mutations in many loci associated with HbA_1c_ (*SPTA1ANK1HK1TMPRSS6*) are known to cause hereditary red blood cell disorders [7] and common variants at several loci (*SPTA1, HFE, ANK1HK1TMPRSS6*) are associated with hematological traits like hemoglobin concentration and mean corpuscular volume [32-34]. Adjustment of models of these common variants predicting HbA_1c_ levels for levels of hemoglobin concentration or mean corpuscular volume attenuate SNP-HbA_1c_ relationships, suggesting mediation of HbA_1c_ varation by elements of erythrocyte biology [7]. Further, a recent genetic association study showed some differences in the genetic regulation of hematological traits in Europeans compared with Africans [35]. Our analyses of differentiation and selection suggest that there may be some selection pressure at the *ANK1HK1ATP11A, TMPRSS6* and *ABC11/G6PC2* loci, the first four of which are erythrocyte-related loci. However, in the present study, race-ethnic differences in association with HbA_1c_ by SNP were observed at only two of these loci (*ANK1* [rs4737009] and *TMPRSS6*). We also examined inter-population allele frequency differences of trait-associated SNPs which may indicate that selection is operating on the trait [36]. While frequencies of some disease-associated alleles have been reported as largely heterogeneous between race-ethnicities [36-39], other data suggest no greater differentiation than would be expected from a random set of SNPs [40]. We found heterogeneous inter-race-ethnic risk allele frequencies at six of the HbA_1c_-associated SNPs and three of these (SNPs near *ANK1* [both SNPs] and *TMPRSS6*) showed inter-race heterogeneity in SNP association with HbA_1c_.

We found modest race-ethnic differences in the association of individual or collective HbA_1c_-associated SNPs and levels of HbA_1c_. We found nominally significant associations with an HbA_1c_-associated SNP genotype score and levels of HbA_1c_ in NHW, as expected, and also in MA, but not in NHB individuals. Ancestral variation in LD probably accounts for some of this difference in association. LD is more fine-grained in genomes of African individuals [22], so some of the HbA_1c_-associated SNPs may be more tightly linked to putative functional alleles in NHW and MA than in NHB. Modest power given the relatively small sample size of NHANES III could also account for the relatively weak association of HbA_1c_ SNPs with HbA_1c_ in each race-ethnic group ( Additional file 1: Table S3). No significant interactions were observed, also possibly due to low power. T2D diagnosis was based on fasting glucose with no OGTT, which may have introduced misclassification in T2D status of study subjects. Furthermore, greater heterogeneity exists in NHB, and this heterogeneity may have influenced variability in HbA_1c_ levels. Since there are no ancestry markers available in NHANES to evaluate genetic heterogeneity within populations, we were unable to evaluate substructure within ethnic groups and, for the purposes of this study, assumed little to no intra-population substructure.

Despite previous epidemiological associations of HbA_1c_ levels with mortality or cardiovascular disease [12-19] and race-ethnic variation in mortality rates in NHANES III, we did not see any evidence of an association of HbA_1c_-associated loci with mortality in any race-ethnic group. If HbA_1c_ is associated with mortality, it is likely to be mediated through HbA_1c_’s association with hyperglycemia and insulin resistance, but many HbA_1c_-associated loci are associated with erythrocyte biology and not hyperglycemia. A lack of association of the HbA_1c_-associated SNPs studied here and cardiovascular disease events has also been shown previously in white cohorts [7]. This unlinking of hyperglycemia from HbA_1c_ biology also has bearing on diabetes screening and diagnosis. Another explanation for a lack of association of the HbA_1c_ genetic risk score with mortality is the lack of statistical power due to small sample size within each ethnicity ( Additional file 1: Table S5). When pooling the entire sample and carrying out an interaction model we also observed no significant genetic risk score x ethnicity interaction on mortality.

Race-ethnic differences in HbA_1c_ levels were observed in the present study and have been shown previously [41-46]. Population differences in HbA_1c_ levels are partly attributable to variability in non-biological factors including race-ethnic differences in lifestyle, socioeconomics, health insurance access or screening intensity [41,44]. Further, there are likely race ethnic differences in non-glycemic biological factors including glycemic level, hemoglobinopathies [30,47-49], iron deficiency anemias [21,48,50-54], and erythrocyte survival [48,55,56]. The data suggest that glycemic control is not the only root cause of inter-race-ethnic differences in HbA_1c_. Although the clinical impact of HbA_1c_ genetics on diabetes detection appears to be modest in whites, at least , whether race-ethnic heterogeneity in HbA_1c_ genetics influences diabetes diagnosis in other race-ethnic groups requires further investigation.

The major strengths of this study include genotyping of all 11 known HbA_1c_-associated SNPs in the nationally representative, multi-race-ethnic NHANES III cohort. The heterogeneity of HbA_1c_–associated SNP frequencies across race-ethnic groups and the limited impact of these SNPs on HbA_1c_ level in NHB individuals underscore the importance of extending association studies and the discovery of causal variants to diverse populations for a comprehensive understanding of HbA_1c_ genetic architecture. As diverse populations become increasingly incorporated into genetic studies for variant detection, inter-race-ethnic variation will likely continue to be revealed, necessitating careful investigation of its sources and significance.

## Conclusions

In NHANES III there is substantial RAF race-ethnic heterogeneity at many HbA_1c_ loci. An 11-HbA_1c_- associated SNP genotype score was subtly different by race-ethnicity and was associated with increase in HbA_1c_ in NHW and MA but not NHB. While the numerous potential sources for this race-ethnic heterogeneity in association with HbA_1c_ require further exploration, the data underscore the importance of extending genetic analysis to non-white populations, especially where they may have impact on guidelines for disease screening, diagnosis or management.

## Disclaimer

The findings and conclusions in this report are those of the author(s) and do not necessarily represent the official position of the Centers for Disease Control and Prevention.

## Competing interests

The author(s) declare that they have no competing interests.

## Authors’ contributions

JLG and JBM drafted the manuscript, and QY, JCF, JD, TL, AY, MHC, KMW, NF, and MJK provided critical discussion during the development of the project as well as review of the draft and final manuscript. BCP and JLV carried out statistical analyses with assistance from QY, JD, TL, AY, MHC. JBM conceived of the study and MJK and JBM provided oversight and supervision for its conduct. All authors read and approved the final manuscript.

## Pre-publication history

The pre-publication history for this paper can be accessed here:

http://www.biomedcentral.com/1471-2350/13/30/prepub

## Supplementary Material

Additional file 1 ** Table S1.** Weighted Risk (HbA_1c_-raising) allele frequencies of 11 HbA_1c_-associated SNPs by race-ethnicity, Third National Health and Nutrition Survey (NHANES III); **Table S2.** Weighted allele and genotype frequencies of 11 HbA_1c_-associated SNPs by race-ethnicity, Third National Health and Nutrition Examination Survey (NHANES III) versus HapMap; **Table S3.** Power calculations for HbA_1c_ at alpha=0.05 and alpha=0.05/11 (Bonferroni corrected) assuming similar effect sizes to those published by Soranzo et al. (2010); **Table S4.** Adjusted mean HbA_1c_ levels (%) and an 8-SNP "non-glycemic" genetic risk score by race-ethnicity, Third National Health and Nutrition Examination Survey (NHANES III); **Table S5.** Power calculations for mortality at alpha=0.05 and alpha=0.05/11 (Bonferroni corrected); **Table S6.** Number of LD blocks in 500 kb regions flanking each SNP (based on Haploview version 4.2, HapMap release 27, build 36, Phases II and III, February 2009) and **Table S7.** Scans for signatures of population differentiation and natural selection in 2Mb regions surrounding 10 SNPs associated with HbA_1c_ in Europeans from Haplotter queries by SNP and queries by locus (2Mb regions). Click here for file
